# *Bombyx mori* C-Type Lectin 16 Inhibits BmNPV Proliferation by Degrading Viral Protein Bm9 via Ubiquitin–Proteasome System

**DOI:** 10.3390/biom16060890

**Published:** 2026-06-17

**Authors:** Xiaoyu Sun, Chunguang Cui, Guangrong Huang, Xiaoli Zou, Shaofang Yu, Xin Du, Xia Xu, Jine Chen, Xingjian He, Yongqiang Wang, Linbao Zhu

**Affiliations:** 1 College of Life Sciences, China Jiliang University, Hangzhou 310018, China; 2 Institute of Sericulture and Tea, Zhejiang Academy of Agricultural Sciences, Hangzhou 310021, China

**Keywords:** C-type lectin 16, BmNPV, Bm9, ubiquitin–proteasome system, antiviral immunity

## Abstract

C-type lectins (CTLs) are proteins with carbohydrate-recognition domains. These macromolecules interact with pathogen components, thereby playing important roles in the immune system. Current studies indicate that silkworm CTLs are involved in *Bombyx mori* nucleopolyhedrovirus (BmNPV) infection. Nevertheless, the molecular mechanisms through which these CTLs affect viral infection remain unclear. In this study, *B. mori C-type lectin 16* (*BmCTL16*) was identified in the silkworm. Its expression was significantly downregulated upon BmNPV infection. Functional assays showed that BmCTL16 overexpression suppressed BmNPV proliferation, whereas its knockdown enhanced BmNPV proliferation. Protein–protein interaction assays confirmed that BmCTL16 interacts with BmNPV protein Bm9 in the cytoplasm. Notably, BmCTL16 promoted the degradation of Bm9 via the ubiquitin–proteasome system. Knockdown of Bm9 by siRNA significantly reduced BmNPV proliferation, confirming that Bm9 is the key target for BmCTL16 to exert its antiviral function. Collectively, this study reveals a novel CTL-mediated antiviral mechanism. BmCTL16 interacts with Bm9 and promotes its ubiquitin–proteasome degradation, thereby inhibiting viral proliferation. Furthermore, BmNPV evades this host defense by downregulating BmCTL16 expression. These findings enhance our understanding of silkworm CTL-mediated antiviral defense and offer novel perspectives on host–virus interactions in *B. mori*.

## 1. Introduction

*Bombyx mori* nucleopolyhedrovirus (BmNPV) belongs to the Baculoviridae family and carries a double-stranded circular DNA genome. It is one of the most harmful viral pathogens in the sericulture industry, causing severe economic losses [1]. Following viral infection, silkworms activate multiple innate immune pathways to defend against the virus. Antiviral proteins present in the midgut digestive juice, including lipase member H-A, contribute to the first line of defense [2]. Furthermore, melanization, autophagy, apoptosis, and non-coding RNA-mediated regulation also contribute to antiviral responses [3,4,5]. Nevertheless, the precise molecular basis underlying silkworm resistance to BmNPV remains largely unknown, and no effective therapeutic agents are available to control BmNPV infection.

C-type lectins (CTLs) are a class of proteins containing C-type lectin-like domains. They are crucial components of the innate immune system and participate in various biological processes [6]. In mammals, CTLs such as surfactant proteins D (SP-D) exhibit antiviral activity by interacting with viral glycoproteins. SP-D binds to the GP120 glycoprotein of HIV-1, suppressing virus-induced cytokine storms and limiting viral transmission [7,8]. Transgenic expression of human CLEC18A in *Aedes aegypti* enhances Toll immune pathway responses and alters gut microbiota composition, reducing dengue virus (DENV) genome copies and viral titers in the midgut by 70% [9]. During viral infection in insects, CTLs can induce protective responses. For instance, *Musca domestica* CTLs (MdCTL-1 and MdCTL-2) inhibited *Autographa californica* multiple nucleopolyhedrovirus (AcMNPV) infection when co-incubated with the virus [10], whereas BmCTL5 was weakly activated by *Bombyx mori* cypovirus (BmCPV) infection, and its knockdown inhibited the JAK/STAT signaling pathway [11]. Several insect CTLs have been reported to exhibit antiviral activity, but how they recognize viruses and which downstream effectors are involved remain unclear.

BmNPV encodes multiple glycoproteins, including the envelope glycoprotein GP64 and the mitochondria-associated glycoprotein GP37, which play important roles in viral adsorption, entry, and proliferation [12,13]. In addition, BmNPV genes such as *Bm9* and *Bm54* have been implicated in the regulation of viral proliferation [14,15]. Whether these BmNPV proteins serve as targets for host CTLs remains to be elucidated.

The ubiquitin–proteasome system (UPS) serves as the main degradation machinery for intracellular proteins. This system functions through a sequential cascade: ubiquitin becomes attached to substrate proteins via the coordinated action of E1 activating enzymes, E2 conjugating enzymes, and E3 ligases. Subsequently, the ubiquitin-tagged proteins are recognized and broken down by the 26S proteasome [16]. The UPS plays a complex dual role in virus–host interactions, serving both as a host antiviral defense mechanism and as a system hijacked by viruses to facilitate replication. For example, during SARS-CoV-2 infection, host E3 ligases target the viral nsp16 protein for ubiquitination and degradation, thereby limiting viral replication [17]. In influenza virus infection, the host employs the UPS for viral protein degradation, whereas viruses have evolved antagonistic strategies [18]. In the context of BmNPV infection, UPS-associated proteins, including PSMD14, PSMA6, and PSMZ, have been shown to be involved in budded virus egress; interfering with their expression affects GP64 secretion and viral release [19]. Moreover, proteasome inhibition by MG132 during the early stage of BmNPV infection significantly reduces budded virus production [20]. These findings indicate that BmNPV depends on the silkworm UPS to complete its life cycle; however, it remains unknown whether host proteins can utilize the UPS to target BmNPV proteins for degradation.

This study investigated the association between BmCTL16 and BmNPV during viral infection. First, the expression changes in BmCTL16 upon BmNPV infection were validated, and its effect on viral proliferation was assessed through gene overexpression and knockdown in both cell-based and larval models. Subsequently, co-immunoprecipitation coupled with mass spectrometry was used to identify BmNPV proteins that bind to BmCTL16, with a focus on Bm9. The interaction mode and the effect of BmCTL16 on the stability of Bm9 were further characterized to determine whether BmCTL16 promotes viral protein degradation via the UPS. This study aims to elucidate a novel antiviral mechanism mediated by a silkworm C-type lectin.

## 2. Materials and Methods

### 2.1. Silkworm Strains, Cell Lines, and Virus

The study utilized fifth-instar larvae of the Nistari silkworm variety maintained under controlled conditions at 25 °C with 70% humidity while being fed fresh mulberry foliage. Purified BmNPV (T3 strain) was kept in our laboratory. *Bombyx mori* ovary cell line (BmN, Cellosaurus accession number CVCL_Z633) and recombinant baculovirus expressing EGFP (BV-EGFP) were acquired from professor Jiaping Xu (Anhui Agricultural University, Hefei, China). BmN cells were maintained at 27 °C in TC-100 medium (Livning, Beijing, China) containing 12% fetal bovine serum (FBS). For virus infection, BmN cells were infected with BV-EGFP at a multiplicity of infection (MOI) of 2, silkworm larvae were orally inoculated 3 μL of purified BmNPV (1 × 10^7^ OBs/mL).

### 2.2. Overexpression and RNA Interference

The coding sequences of *BmCTL16* (GenBank XM_062672351.1) and *Bm9* (GenBank NC_001962.1; nucleotides 8725–9357) were amplified from cDNA of BmNPV-infected silkworm larvae. Insertion of the PCR products into linearized pIZ-Flag or pIZ-Myc vectors using 2 × Assembly Mix homologous recombination enzyme (TransGen, Beijing, China), generating pIZ-BmCTL16-Flag, pIZ-Bm9-Flag, pIZ-BmCTL16-Myc, and pIZ-Bm9-Myc. Transfection was performed using the DNA transfection reagent (Neofect, Beijing, China), using 2 µg of plasmid per 1 mL of culture medium. Overexpression efficiency was examined at 48 h post-transfection. The siRNAs targeting *BmCTL16* and *Bm9* were synthesized by Sangon Biotech Co., Ltd. (Shanghai, China). For gene knockdown in BmN cells, siRNA transfection was performed using RNA TransMate Reagent (Sangon, Shanghai, China). For in vivo RNA interference, dsRNA was prepared using the TranscriptAid T7 High Yield Transcription Kit (Thermo Fisher Scientific, Vilnius, Lithuania). The purified dsRNA (5 µg per larva) was microinjected into the hemocoel of silkworm larvae. Primers used for PCR and dsRNA preparation were listed in Table S1. The siRNA sequences are listed in Table S2.

### 2.3. Real-Time PCR

To determine the transcriptional levels of target genes, total RNA was extracted and reverse-transcribed, and real-time PCR was carried out using SYBR Green Master Mix (TOYOBO, Osaka, Japan), following a previously described protocol [5]. With normalization to the control group, the relative gene expression levels were determined using the 2^−ΔΔCt^ method. To evaluate viral proliferation, genomic DNA was extracted from BmNPV-infected silkworm larvae or BmN cells by phenol–chloroform extraction. Real-time PCR was performed using the extracted DNA as a template to quantify the copy number of the viral *VP39* gene, and all primers are listed in Table S3.

### 2.4. Protein Expression, Purification, and Antibody Preparation

The coding sequences of *BmCTL16* and *VP39* were amplified with specific primers (Table S1) and ligated into linearized pET-30a vector using 2× Assembly Mix homologous recombination enzyme (TransGen, Beijing, China) to generate pET-30a-BmCTL16 and pET-30a-VP39. Recombinant BmCTL16 and VP39 proteins were expressed and purified as described previously [4]. These recombinant proteins were sent to HuaAn Biotechnology Co., Ltd. (Hangzhou, China) for the preparation of polyclonal antibodies (IgG) against BmCTL16 and VP39.

### 2.5. Western Blot

The primary antibodies used in this study included anti-Flag (1:500), anti-Myc (1:400), anti-β-tubulin (1:5000), HRP-conjugated anti-mouse IgG (1:10,000), and HRP-conjugated anti-rabbit IgG (1:10,000) were commercially purchased from TransGen (Beijing, China). Anti-Ub (1:500) and anti-Na^+^/K^+^ ATPase (1:500) were purchased from Beyotime (Shanghai, China). Anti-BmCTL16 (1:200) and anti-VP39 (1:200) were generated in-house. Protein separation, transfer, immunoblotting, and signal detection were carried out following a standard protocol [5]. After detection, the membrane was stripped using a stripping buffer (Beyotime, Shanghai, China) to remove bound antibodies and subjected to the same analysis procedure for the next target. All original Western blot images are available in the Supplementary Material (Figure S1).

### 2.6. Analysis of the Localization and Extracellular Antiviral Function of BmCTL16

To determine the localization of BmCTL16, BmCTL16-Flag was expressed in BmN cells. The cell lysates and culture supernatants were analyzed by anti-Flag IP and Western blotting. Cytoplasmic and membrane fractions were separated using Membrane and Cytosol Protein Extraction Kit (Beyotime, Shanghai, China) and examined by Western blotting. To determine whether extracellular BmCTL16 affects viral proliferation, two parallel approaches were used: neutralization of secreted BmCTL16 with specific antibodies and supplementation with excess recombinant BmCTL16 protein. After treatment, viral proliferation was assessed by real-time PCR and fluorescence microscopy. For antibody neutralization, based on the method described [21], with modification, purified rabbit anti-BmCTL16 IgG was diluted in TC-100 medium to 25 μg/mL, BmN cells were then cultured in this medium and infected with BmNPV. For protein supplementation, based on the physiological concentrations reported for C-type lectins in insects (approximately 21 μg/mL in immune-induced *Heliothis virescens* pupal hemolymph) and humans Mannose-binding lectin (up to 10 μg/mL for serum MBL) [22,23], the medium was supplemented with recombinant BmCTL16 protein to a final concentration of 50 μg/mL, and infected with BmNPV simultaneously.

### 2.7. Assessment of Viral Proliferation

To evaluate viral proliferation, three complementary approaches were employed. For quantification of viral DNA accumulation, both infected BmN cells and the culture supernatant were collected together after infection. Total DNA was extracted from the combined samples, and the copy number of the viral *VP39* gene was quantified by real-time. For analysis of viral protein level, total protein of infected BmN cells was extracted. The level of VP39 protein was assessed by Western blot with the anti-VP39 antibody. For visualization of viral infection, viral fluorescence was observed and imaged using a fluorescence microscope.

### 2.8. Immunofluorescence Staining

BmN cells were fixed with Immunostaining Fixative (Beyotime, Shanghai, China) for 0.5 h and subjected to permeabilization with Immunostaining Permeabilization Buffer (Beyotime, Shanghai, China). After blocking with QuickBlock™ Protein-Free Blocking Buffer (Beyotime, Shanghai, China), anti-Flag-AF555 (Aladdin, Shanghai, China) and anti-Myc-AF488 (Aladdin, Shanghai, China) were used to incubate the cells. Following washes with PBS, nuclei were counterstained with DAPI solution (Beyotime, Shanghai, China) for 5 min. After a final wash, the slides were mounted and examined under a laser scanning confocal microscope.

### 2.9. Co-Immunoprecipitation (Co-IP) Coupled with LC-MS/MS

To identify viral proteins interacting with BmCTL16, cells transfected with pIZ-BmCTL16-Flag were collected and lysed in Western and IP lysis buffer (Beyotime, Shanghai, China) containing protease inhibitor. The supernatant obtained after centrifugation was incubated with anti-Flag beads (Biolinkedin, Shanghai, China). The beads were then washed with lysis buffer three times to remove impurities, and the bound proteins were eluted with glycine buffer (0.1 M, pH 2.5). The proteins obtained after immunoprecipitation were subjected to LC-MS/MS analysis for comprehensive identification of all protein components within the sample. Cells overexpressing EGFP-Flag were processed in parallel under identical conditions as a control to exclude non-specific binding proteins, and the remaining specific interactors were identified as candidate BmCTL16-interacting proteins. To further verify the protein–protein interaction, BmN cells were co-transfected with pIZ-BmCTL16-Myc together with pIZ-Bm9-Flag. pIZ-EGFP-Myc served as a reference for excluding non-specific interactions. The samples were then subjected to Co-IP and Western blot analysis.

### 2.10. Protein Structure Prediction and Docking

The structure of BmCTL16 was predicted using the SWISS-MODEL server. Templates with the highest sequence identity and coverage was selected for model building, and the resulting models were evaluated using the global model quality estimation (GMQE) score function. Models with a GMQE score greater than 0.75 were considered reliable and used for subsequent analysis. For Bm9, which lacked suitable homologous templates, the structure was predicted using AlphaFold 3. The monomeric model was assessed by pLDDT, with the majority of residues scoring >90, indicating high confidence. Molecular docking between BmCTL16 and Bm9 was performed using the GRAMM server. The resulting docking model was submitted to the PDBePISA server for interface analysis, where key interacting residues, hydrogen bonds, and salt bridges were identified. These binding interfaces and residues were subsequently visualized and rendered using PyMOL (version 3.1.6.1).

### 2.11. Transcriptomic Analysis

To identify potential UPS components regulated by BmCTL16, BmN cells were divided into three groups: Control (treated with transfection reagent only), BmCTL16 knockdown (transfected with si-BmCTL16), and BmCTL16 overexpression (transfected with pIZ-BmCTL16-Flag). At 48 h post-transfection, total RNA was extracted, and RNA was enriched using Oligo(dT) magnetic beads. Libraries were prepared and sequenced on an Illumina platform. Raw reads were filtered and mapped to the *B. mori* reference genome using HISAT2 (version 2.2.1). Gene expression was quantified as FPKM. Differential expression analysis was performed, and genes with *p*adj < 0.05 were considered significant. Genes downregulated in the BmCTL16 knockdown group and upregulated in the BmCTL16 overexpression group were identified.

### 2.12. Statistical Analysis

Each experiment was performed with three independent replicates (*n* = 3 per group). Data were analyzed using Student’s *t*-test and ANOVA in GraphPad Prism 8 (GraphPad Software, San Diego, CA, USA). Results were expressed as mean ± SD. Asterisk indicates significant differences (* *p* < 0.05, ** *p* < 0.01).

## 3. Results

### 3.1. Tissue Expression Pattern and BmNPV-Induced Regulation of BmCTL16

**Figure 1:**
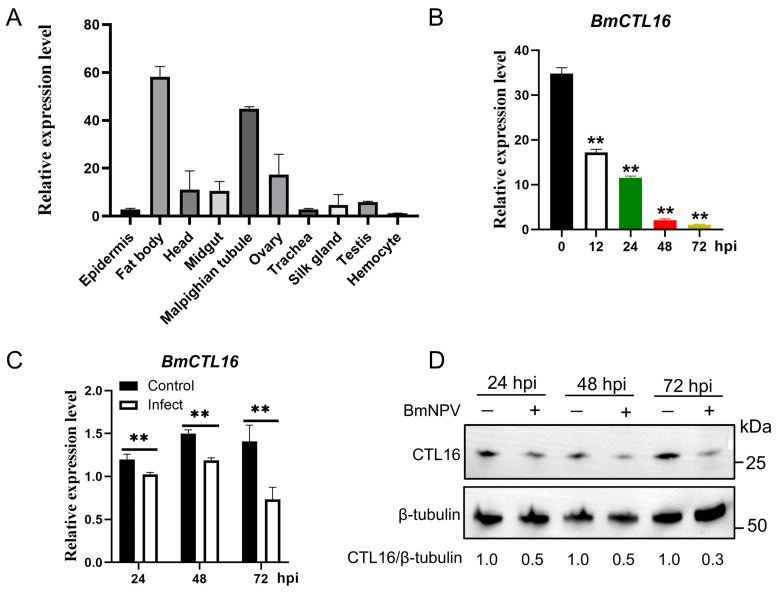
Real-time PCR analyzed the tissue expression pattern and BmNPV-induced regulation of *BmCTL16*. (**A**) Relative expression levels of *BmCTL16* in silkworm different tissues. (**B**) BmNPV-modulated BmCTL16 expression in BmN cells at 12–72 hpi. (**C**) Transcript and (**D**) protein levels of BmCTL16 in the silkworm larval fat body following BmNPV infection at 24–72 hpi. Numbers below bands indicate BmCTL16/β-tubulin grayscale ratios. (Original western blot images see Supplementary Material). Results were expressed as mean ± SD. Asterisk indicates significant differences (** *p* < 0.01).

Prior transcriptomic and proteomic profiling of BmNPV-infected silkworm [24,25] identified BmCTL16 as a responsive gene whose transcript and protein abundance markedly downregulated following viral infection (Table S4). To validate these findings, real-time PCR was used to analyze the tissue-specific and BmNPV-induced expression patterns of *BmCTL16*. Under basal conditions, the fat body exhibited significantly higher *BmCTL16* expression than other tissues (Figure 1A). Following BmNPV infection, *BmCTL16* transcript levels showed a progressive decline from 12 to 72 h post-infection (hpi) in BmN cells (Figure 1B). Endogenous BmCTL16 expression in the fat body was significantly reduced at both the transcript (Figure 1C) and protein (Figure 1D) levels from 24 to 72 hpi.

### 3.2. BmCTL16 Overexpression Inhibits BmNPV Proliferation In Vitro

**Figure 2:**
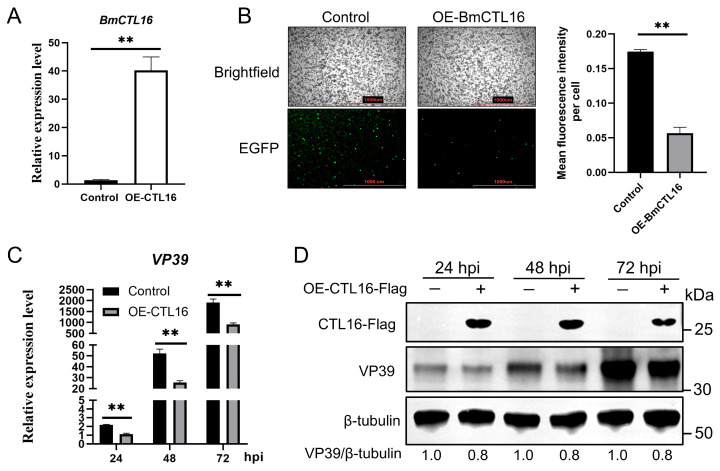
BmCTL16 overexpression inhibits BmNPV proliferation in vitro. (**A**) Overexpression efficiency of BmCTL16 was analyzed by real-time PCR. (**B**) Viral fluorescence intensity in BmN cells overexpressing BmCTL16 was observed by fluorescence microscopy at 48 hpi. (**C**) Real-time PCR analyzed the BmNPV proliferation upon overexpressing BmCTL16. (**D**) Western blot analyzed the VP39 protein levels in BmN cells overexpressing BmCTL16. Numbers below bands indicate VP39/β-tubulin grayscale ratios. (Original western blot images see Supplementary Material). Results were expressed as mean ± SD. Asterisk indicates significant differences (** *p* < 0.01).

To investigate the effect of BmCTL16 on viral proliferation, BmN cells were transfected with the pIZ-BmCTL16-Flag plasmid or the empty vector control (Figure 2A). Following infection with BV-EGFP, fluorescence microscopy at 48 hpi revealed a significantly lower density of the green fluorescent signal in cells overexpressing BmCTL16 compared to controls (Figure 2B). Accordingly, analysis of viral *VP39* gene copies and VP39 protein levels showed a significant decrease from 24 to 72 hpi in cells overexpressing BmCTL16 (Figure 2C,D). These findings indicate that BmCTL16 overexpression inhibits BmNPV proliferation in vitro.

### 3.3. BmCTL16 Knockdown Enhances BmNPV Proliferation In Vitro and In Vivo

**Figure 3:**
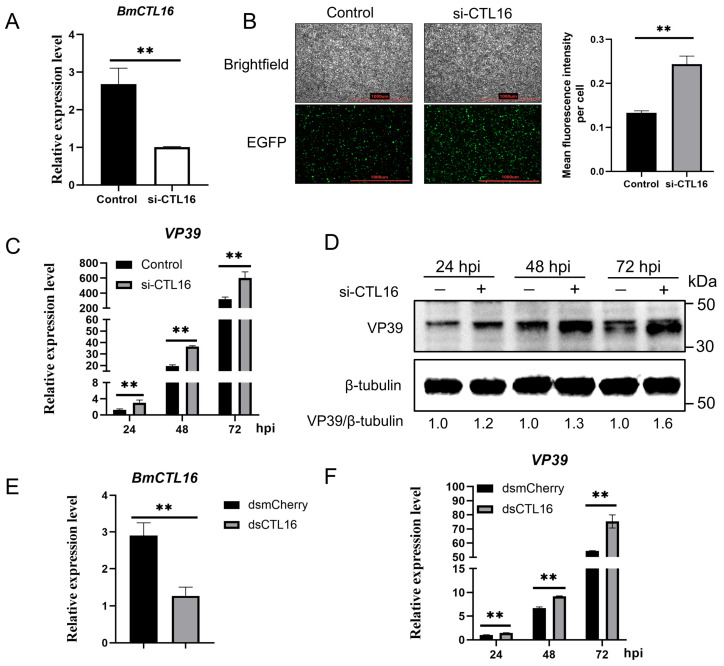
BmCTL16 knockdown enhances BmNPV proliferation in vitro and in vivo. (**A**) siRNA efficiency of BmCTL16 in BmN cells was analyzed by real-time PCR. (**B**) The effect of BmCTL16 knockdown on BV-EGFP fluorescence in BmN cells was observed at 48 hpi. (**C**) BmNPV *VP39* gene copies were measured by real-time PCR in BmN cells transfected with siRNA-BmCTL16 to assess viral proliferation. (**D**) BmNPV VP39 protein levels were measured by Western blot in BmN cells transfected with siRNA-BmCTL16 to assess viral proliferation. Numbers below bands indicate VP39/β-tubulin grayscale ratios. (Original western blot images see Supplementary Material). (**E**) Knockdown efficiency of BmCTL16 in the fat body was analyzed by real-time PCR. (**F**) Effect of BmCTL16 knockdown on *VP39* gene copies in the larval fat body was analyzed by real-time PCR. Results were expressed as mean ± SD. Asterisk indicates significant differences (** *p* < 0.01).

To further investigate the function of BmCTL16, siRNA was used in BmN cells and dsRNA in silkworm larvae to knock down its expression. siRNA transfection efficiently reduced BmCTL16 expression in BmN cells (Figure 3A), after BV-EGFP infection, these cells exhibited a notable increase in green fluorescence at 48 hpi compared to control (Figure 3B). Accordingly, both *VP39* gene copies and VP39 protein levels were significantly elevated in BmCTL16-knockdown cells from 24 to 48 hpi (Figure 3C,D). In silkworm larvae, following confirmation of knockdown efficiency (Figure 3E), BmCTL16-specific dsRNA was injected into silkworm larvae, which were subsequently infected with BmNPV. Real-time PCR analysis confirmed that viral *VP39* gene copies in the fat body were significantly higher in dsRNA-treated larvae (Figure 3F). These results demonstrate that reducing BmCTL16 expression promotes BmNPV proliferation in vitro and in vivo. Taken together with the overexpression data, our findings indicate that BmCTL16 plays a key role in silkworm antiviral defense against BmNPV.

### 3.4. BmCTL16 Exerts Antiviral Effect Exclusively Inside the Cell Instead of Outside

**Figure 4:**
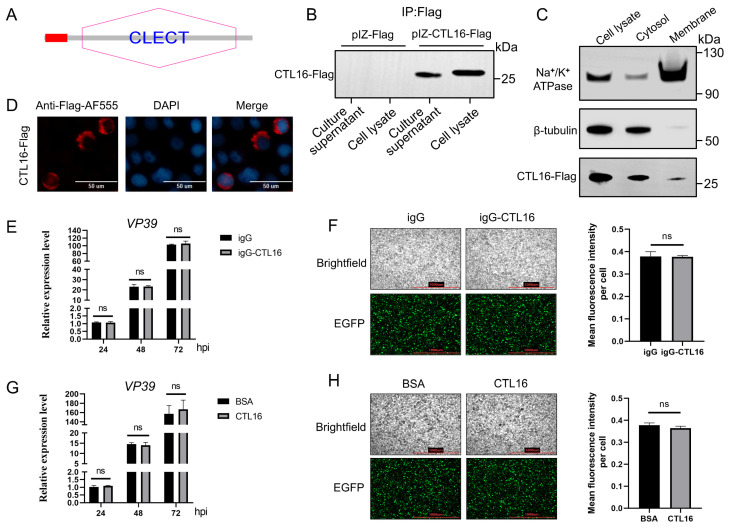
BmCTL16 exerts antiviral effect exclusively inside the cell instead of outside. (**A**) Structural domains of BmCTL16 predicted by SMART (https://smart.embl.de; accessed on 20 April 2026). The red box indicates the signal peptide, and the shaded hexagon represents the C-type lectin-like domain (CTLD). (**B**) At 48 h post-transfection, BmCTL16-Flag was enriched from culture supernatant and whole-cell lysate using anti-Flag beads and analyzed by Western blot. (**C**) Cytoplasmic (Cytosol) and membrane-enriched (Membrane, containing Golgi apparatus, ER, mitochondria and other organelle membranes as well as their luminal contents) fractions were analyzed by Western blotting for BmCTL16 distribution. (Original western blot images see Supplementary Material). (**D**) BmN cells were transfected with pIZ-BmCTL16-Flag, stained with anti-Flag-AF555 and DAPI, and examined by confocal microscopy. (**E**) *VP39* DNA copy numbers were measured by real-time PCR in BmN cells that had been treated with anti-BmCTL16 IgG. (**F**) BV-EGFP fluorescence in anti-BmCTL16 IgG treated BmN cells was observed at 48 hpi. (**G**) *VP39* DNA copies were analyzed by real-time PCR in BmN cells treated with exogenous recombinant BmCTL16 protein. (**H**) BV-EGFP fluorescence in recombinant BmCTL16 protein treated BmN cells was observed at 48 hpi. Results were expressed as mean ± SD. ns, not significant.

Bioinformatic analysis revealed BmCTL16 encodes a protein containing a canonical C-type lectin-like domain (CTLD) and an N-terminal signal peptide (Figure 4A). To determine the localization of BmCTL16, BmCTL16-Flag was expressed in BmN cells. The results confirmed BmCTL16 presence in these extracellular and intracellular fractions (Figure 4B). Subcellular fractionation followed by Western blotting showed that BmCTL16 was predominantly localized in the cytoplasm (Figure 4C). This was further confirmed by immunofluorescence analysis of intracellular BmCTL16 (Figure 4D). Therefore, to evaluate whether extracellular BmCTL16 influences viral infection, anti-BmCTL16 IgG or recombinant BmCTL16 protein (expressed and purified in *E. coli*; Figure S2) was added to BmN cell culture to neutralize secreted BmCTL16 or to provide an exogenous source, respectively. Results showed that anti-BmCTL16 IgG treatment and exogenous addition of recombinant BmCTL16 protein had no effect on BmNPV proliferation (Figure 4E–H), indicating that BmCTL16 exerts antiviral effect exclusively inside the cell instead of outside.

### 3.5. BmCTL16 Interacts with BmNPV Protein Bm9

**Figure 5:**
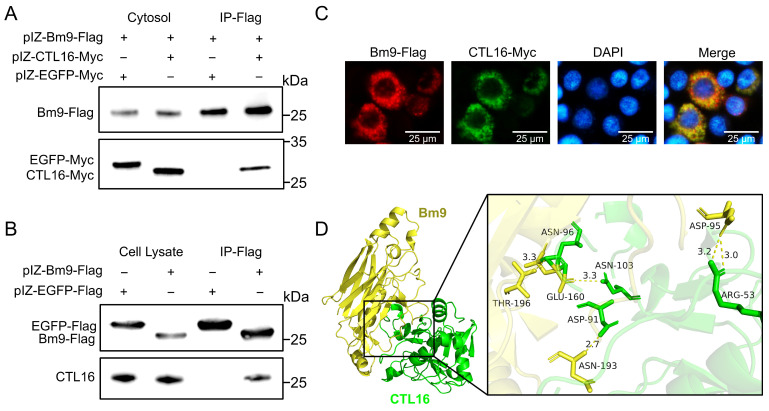
BmCTL16 interacts with BmNPV protein Bm9. (**A**) Co-IP assays were performed on the cytoplasmic fraction of BmN cells co-expressing BmCTL16-Myc and Bm9-Flag. (**B**) Interaction between endogenous BmCTL16 and Bm9 was examined by Co-IP in BmNPV-infected BmN cells. (Original western blot images see Supplementary Material). (**C**) Subcellular colocalization of BmCTL16 with Bm9 was visualized by immunofluorescence. BmN cells co-expressing BmCTL16-Myc and Bm9-Flag were stained with anti-Myc-AF488, anti-Flag-AF555 and DAPI. (**D**) Molecular docking simulations predicted the binding interfaces between BmCTL16 and GP37. Key residues involved in the interactions are indicated.

Based on the intracellular antiviral activity of BmCTL16, its molecular mechanism was further investigated by identifying potential viral interaction proteins. BmN cells overexpressing BmCTL16-Flag were infected with BmNPV. Co-IP coupled with LC-MS/MS identified GP37 and Bm9 as candidate interacting proteins of BmCTL16 after subtracting proteins common to the control (Table S5). As Bm9 plays an essential role in viral proliferation [14], it was selected for subsequent analyses. To verify the interactions with BmCTL16 and Bm9, BmN cells were co-transfected with plasmids expressing BmCTL16-Myc and Bm9-Flag. Co-IP assays performed on the cytoplasmic fraction revealed that BmCTL16 interacts with Bm9 in the cytoplasm (Figure 5A). Endogenous BmCTL16 was also discovered to interact with Bm9 (Figure 5B). Fluorescence co-localization assays showed that BmCTL16 and Bm9 co-localized in the cytoplasm (Figure 5C). Molecular docking simulations performed after homology modeling predicted a stable binding complex between BmCTL16 and Bm9. Key residues forming the potential interaction interface were predicted (Figure 5D). Together, these results verify that BmCTL16 directly interacts with the BmNPV protein Bm9 in the cytoplasm.

### 3.6. BmCTL16 Promotes Degradation of Bm9 via the Ubiquitin–Proteasome System

**Figure 6:**
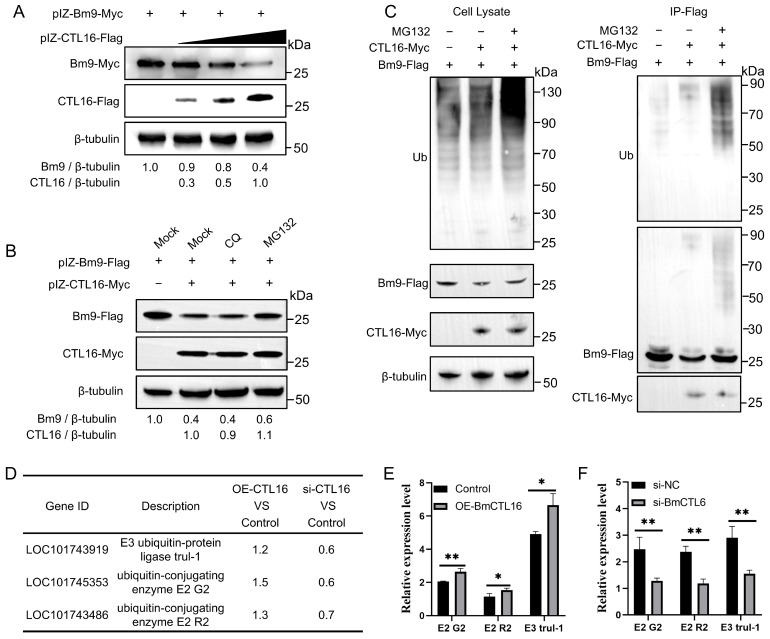
BmCTL16 promotes degradation of Bm9 via the ubiquitin–proteasome system. (**A**) BmN cells were co-transfected with Bm9-Myc plasmid (at a constant amount of 1 μg/mL) and BmCTL16-Flag plasmid (0, 0.5, 1, and 2 μg/mL). (**B**) BmN cells were co-transfected with Bm9-Flag and BmCTL16-Myc plasmids (1.5 μg/mL each). Cells were then treated with 40 μM CQ or 5 μM MG132. Numbers below bands indicate Bm9 or CTL16/β-tubulin grayscale ratios. (**C**) BmN cells were transfected with Bm9-Flag alone, Bm9-Flag plus BmCTL16-Myc, or Bm9-Flag plus BmCTL16-Myc followed by MG132 treatment. Whole cell lysates and anti-Flag immunoprecipitates (IP-Flag) were analyzed by Western blotting with the indicated antibodies. (Original western blot images see Supplementary Material). (**D**) Transcriptomic analysis showing the expression changes in *UBE2G2*, *UBE2R2*, and *E3 Trul-1* in BmN cells after BmCTL16 overexpression or knockdown. (**E**,**F**) Real-time PCR analysis of *UBE2G2*, *UBE2R2*, and *E3 Trul-1* expression in BmN cells after *BmCTL16* overexpression (**E**) or knockdown (**F**). Results were expressed as mean ± SD. Asterisk indicates significant differences (* *p* < 0.05, ** *p* < 0.01).

Degradation of viral proteins is a common antiviral strategy. To determine whether the interaction between BmCTL16 with Bm9 affects the stability of viral proteins, protein degradation assays were performed. In cells co-expressing BmCTL16-Myc with Bm9-Flag, elevated BmCTL16 expression caused a dose-dependent reduction in Bm9 protein levels (Figure 6A), indicating that BmCTL16 promotes Bm9 degradation. Degradation of cellular proteins mainly proceeds via the autophagic pathway or the ubiquitin–proteasome system (UPS). To identify the degradation pathway involved, cells co-expressing BmCTL16 and Bm9 were treated with the autophagy inhibitor (CQ, chloroquine) or the proteasome inhibitor (MG132). The findings indicated that the degradation of Bm9 by BmCTL16 was blocked by MG132 but not by CQ (Figure 6B). To confirm whether BmCTL16 promotes ubiquitination of Bm9, immunoprecipitation of Bm9-Flag from transfected BmN cells was performed, followed by Western blotting. The results show that MG132 treatment markedly increased total ubiquitination signals in whole cell lysates. After anti-Flag IP, polyubiquitinated Bm9 species were detected upon co-expression of BmCTL16, and these signals were further accumulated by MG132 treatment (Figure 6C), indicating that BmCTL16 facilitates Bm9 ubiquitination and subsequent proteasomal degradation. To explore potential UPS components that may be involved, transcriptomic analyses were performed in BmN cells with BmCTL16 overexpression or knockdown. Genes that showed consistent expression changes positively correlated with BmCTL16 levels were identified (Table S6). Among them, several UPS-related genes, including *ubiquitin-conjugating enzyme E2 G2*, *ubiquitin-conjugating enzyme E2 R2*, and the *E3 ubiquitin-protein ligase trul-1* were upregulated upon BmCTL16 overexpression and downregulated upon BmCTL16 knockdown (Figure 6D). These changes were validated by real-time PCR (Figure 6E,F). Together with the inhibitor experiments, these results demonstrate that BmCTL16 promotes degradation of Bm9 via ubiquitin–proteasome system.

### 3.7. Functional Analysis of Bm9 in BmNPV Proliferation

**Figure 7:**
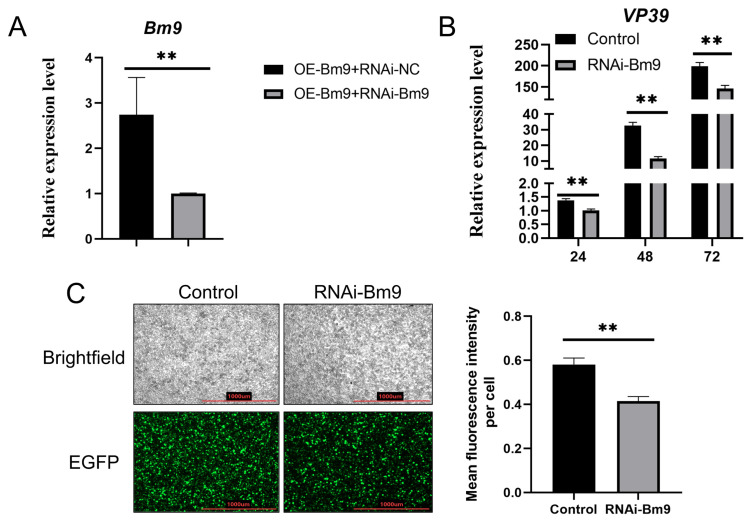
Effects of Bm9 knockdown on BmNPV proliferation. (**A**) *Bm9* siRNA knockdown efficiency. BmN cells were co-transfected with Bm9-Flag plasmid and either si-Bm9 or si-NC. The knockdown efficiency of si-Bm9 was measured by real-time PCR. (**B**) Effect of *Bm9* knockdown on viral DNA replication. BmN cells transfected with si-Bm9 were infected with BmNPV, and *VP39* gene copies were quantified by real-time PCR. (**C**) The effect of *Bm9* knockdown on viral fluorescence was examined at 48 hpi. Results were expressed as mean ± SD. Asterisk indicates significant differences (** *p* < 0.01).

To determine whether Bm9 affects BmNPV proliferation, RNA interference was employed to suppress *Bm9* expression during viral infection, and the effects on viral proliferation were examined. Real-time PCR analysis showed that transfection with Bm9-specific siRNA significantly reduced *Bm9* transcript levels (Figure 7A). *Bm9* knockdown led to a significant decrease in *VP39* gene copies from 24 to 72 hpi (Figure 7B) and a marked reduction in viral fluorescence intensity at 48 hpi (Figure 7C). These results indicate Bm9 serves a key function in BmNPV proliferation, which is consistent with previous reports [14].

## 4. Discussion

C-type lectins are important components of the innate immune system and play key roles in antiviral defense in insects. Our study provides several lines of evidence supporting the antiviral role of BmCTL16 during BmNPV infection. Insect fat body is an important organ for insect immunity and metabolism, producing a variety of immune-related proteins, including pattern recognition receptors and antimicrobial peptides, and contributes significantly to the defense against invading pathogens [26]. Our results revealed that BmCTL16 was abundantly expressed in the fat body (Figure 1A), suggesting its potential involvement in silkworm immune responses. More importantly, BmNPV infection induced a time-dependent downregulation of BmCTL16 expression (Figure 1B–D). This expression pattern differs from those of other silkworm CTL proteins in response to viral infection. For instance, BmCTL5 was only weakly activated upon BmCPV infection [11]. These results suggest that different CTLs respond differently to viral infections, likely reflecting their specialized roles in host defense. Viruses often evade host immunity by downregulating or degrading host antiviral factors [6,27]. This phenomenon is also prevalent in insect virus–host interactions. For instance, during rice gall dwarf virus (RGDV) infection of its leafhopper vector, the viral protein Pns11 binds to the transcription factor Dorsal and blocks its function, thereby decreasing the antiviral protein MxL1 and facilitating viral escape from insect immunity [28]. Single-nucleus transcriptomics of the infected silkworm midgut revealed that the virus reduces the levels of host antiviral factors, including Bmlipase-1 and BmSP142 [29]. Similarly, BmNPV-induced reduction in BmCTL16 could serve as an additional immune escape strategy.

In both cultured cells and silkworm larvae, overexpression and knockdown experiments confirmed the antiviral function of BmCTL16 (Figure 2 and Figure 3), consistent with reports of antiviral functions in other insect CTL proteins [9,10]. Our results showed that BmCTL16 was present in both the cytoplasmic fraction and the culture medium (Figure 4), suggesting that this protein may exist in two localization forms. Similar phenomena have been observed in other species. Ladderlectin, a C-type lectin from large yellow croaker, also contains a signal peptide but localizes in both the cytoplasmic and nuclear compartments [30]. Ec-CTLP, a C-type lectin-like protein from *Epinephelus coioides*, possesses an N-terminal signal peptide yet is exclusively distributed in the cytoplasm [31]. These cross-species observations further support the existence of a subset of CTL family members that do not follow the classical secretory pathway. Notably, although BmCTL16 contains a signal peptide and can be detected in the culture supernatant, neither neutralization of extracellular BmCTL16 nor supplementation with recombinant protein affected viral proliferation (Figure 4), indicating that its antiviral function is primarily exerted inside the cell.

Protein–protein interaction assays confirmed that BmCTL16 interacts with BmNPV protein Bm9, in the cytoplasm (Figure 5). Direct virus–host protein interactions have been reported in other insect virus systems. For example, mosGCTL-7 in mosquitoes is a secreted CTL that binds the JEV envelope protein in the extracellular space and facilitates viral entry [21]. In contrast, BmCTL16 interacts with BmNPV proteins inside the cell, and this intracellular interaction likely serves as the basis for their subsequent degradation. Functional analysis confirmed that BmCTL16 caused a ubiquitination-mediated and proteasome-dependent decrease in Bm9 levels (Figure 6). Due to experimental constraints, the degradation and ubiquitination assays in this study relied primarily on overexpression systems, which may not fully recapitulate endogenous conditions. Nevertheless, this conclusion is indirectly supported by multiple other findings within the same study, including the antiviral effects of BmCTL16, the endogenous interaction with Bm9, and the essential role of Bm9 in viral replication.

The UPS has a dual function in virus–host interactions: it enables host degradation of viral proteins but can also be exploited by viruses to support their replication [32]. Recent studies have revealed the importance of UPS in BmNPV infection. Proteasome inhibition by MG132 during the early stage of infection markedly reduces budded virus production and polyhedrin expression, indicating that UPS is essential for the viral life cycle [20]. Moreover, specific UPS components, including PSMD14, PSMA6, and PSMZ, are required for budded virus egress and GP64 secretion, further demonstrating that BmNPV actively utilizes the host UPS to support its replication [19]. These studies suggest that BmNPV relies on the silkworm UPS to complete its life cycle. Our study reveals that hosts can also utilize UPS to target viral proteins for degradation, a strategy observed across diverse hosts. During SARS-CoV-2 infection, host E3 ligases UBR5 target nsp16 for ubiquitination and degradation, and in PRRSV infection, TRIM29 targets nsp11 for proteasomal degradation, counteracting its suppression of interferon-β [33]. The present study extends this mechanism to the CTL family, suggesting that BmCTL16 may function as an adaptor directing viral proteins to the UPS. Transcriptomic and real-time PCR analyses identified several UPS-related genes that correlated with BmCTL16 expression (Figure 6D–F). However, their direct involvement in Bm9 degradation, and the precise molecular details of how BmCTL16 recruits viral proteins to the ubiquitination machinery, remain to be elucidated.

Knockdown of *Bm9* significantly reduced viral proliferation, as reflected by decreased *VP39* gene copies and viral fluorescence (Figure 7). Our data are fully consistent with previous studies demonstrating that Bm9 plays a critical role in BV production. Deletion of *Bm9* reduced BV yields by more than 10-fold [14]. Moreover, the homolog AcMNPV ortholog AC17 was shown to be a structural component of BV that accelerates viral gene expression upon entry, and its deletion similarly impairs BV production [34]. Thus, the essential role of Bm9 in viral proliferation is well established and provides a mechanistic basis for the antiviral effect of BmCTL16: degradation of Bm9 directly compromises BV production and thereby restricts viral infection.

## 5. Conclusions

**Figure 8:**
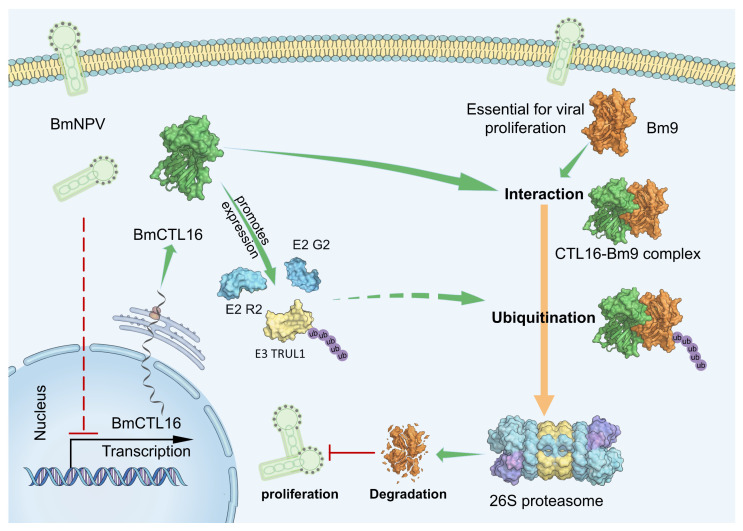
The proposed antiviral mechanism of BmCTL16 effect on BmNPV proliferation. BmCTL16 physically interacts with viral proteins Bm9, facilitating its ubiquitination and proteasome-mediated degradation. Degradation of Bm9 blocks viral proliferation. BmNPV counteracts this host defense by downregulating BmCTL16 expression.

In summary, the antiviral mechanism of BmCTL16 during BmNPV infection was systematically elucidated in this study. Specifically, BmCTL16 interacts with Bm9, leading to Bm9 degradation via the ubiquitin–proteasome system. Degradation of Bm9, a critical viral protein, significantly suppresses viral proliferation. Notably, BmNPV infection downregulates BmCTL16 expression as a viral immune evasion strategy to counteract host antiviral defenses (Figure 8).

## Data Availability

The original contributions presented in this study are included in the article/Supplementary Materials. Further inquiries can be directed to the corresponding authors.

## Supplementary Materials

The following supporting information can be downloaded at: https://www.mdpi.com/article/10.3390/biom16060890/s1, Table S1: List of the primers used in this study for plasmid and dsRNA construction; Table S2: List of siRNA sequence; Table S3: List of the primers used in this study for real-time PCR; Table S4: Transcriptomic and proteomic analysis of BmCTL16 expression upon BmNPV infection; Table S5: Liquid Chromatography–Tandem Mass Spectrometry data for screening BmNPV proteins interacting with BmCTL16; Table S6: List of genes positively correlated with BmCTL16 expression identified by transcriptomic analysis; Figure S1: Original Images for Western blots; Figure S2: Expression and purification of recombinant BmCTL16.
