# Supplementary material for: Bombyx mori C-Type Lectin 16 Inhibits BmNPV Proliferation by Degrading Viral Protein Bm9 via Ubiquitin–Proteasome System
Source: Biomolecules. 2026 Jun 17;16(6):890. doi: 10.3390/biom16060890 (PMC13297094; doi:10.3390/biom16060890)

Original Images for Westen Blots Fig 1 D

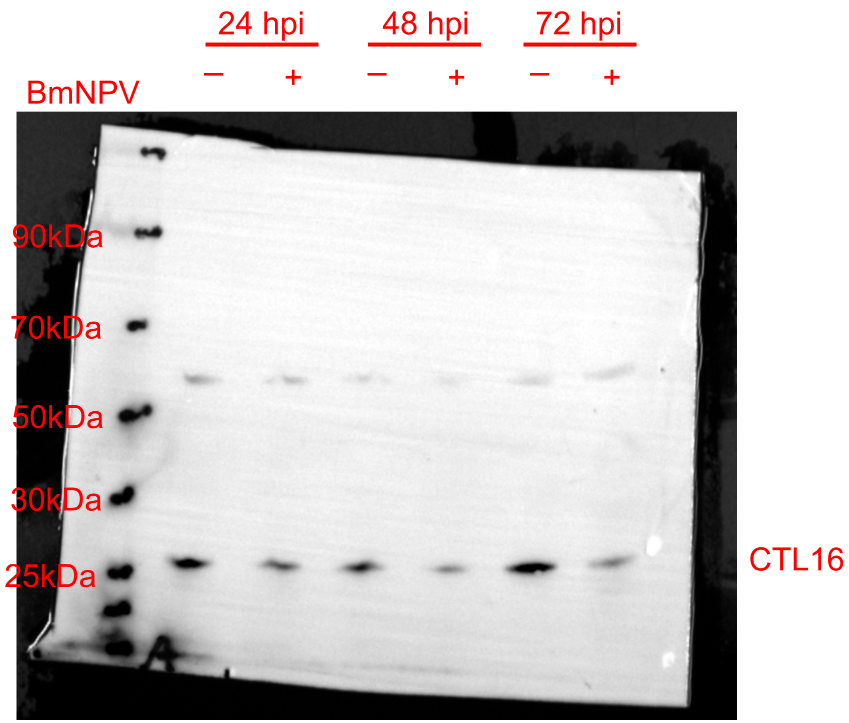

Anti-CTL16

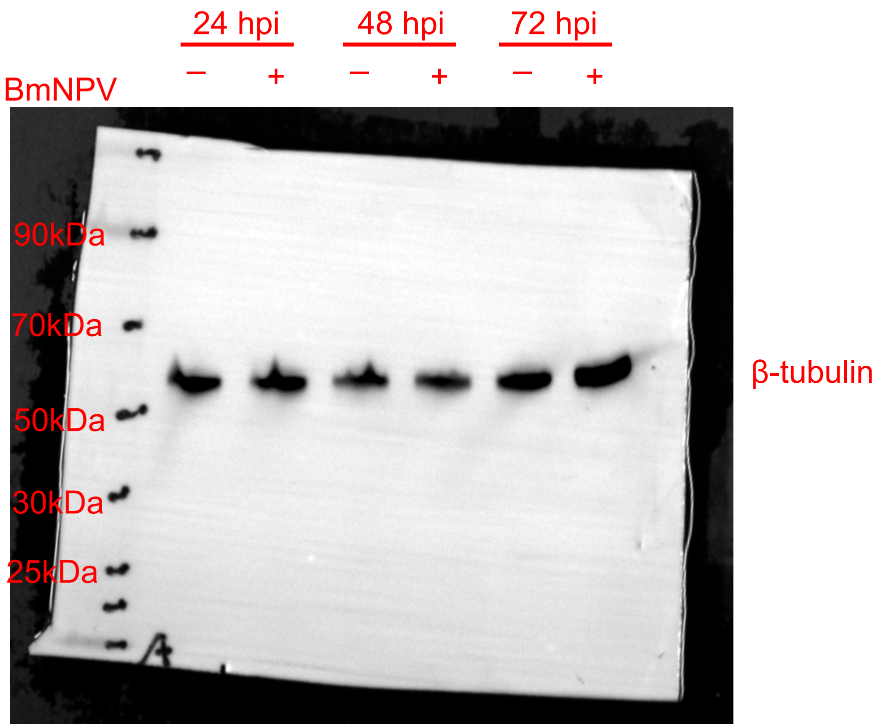

Anti-β-tubulin

Original Images for Westen Blots Fig 2 D

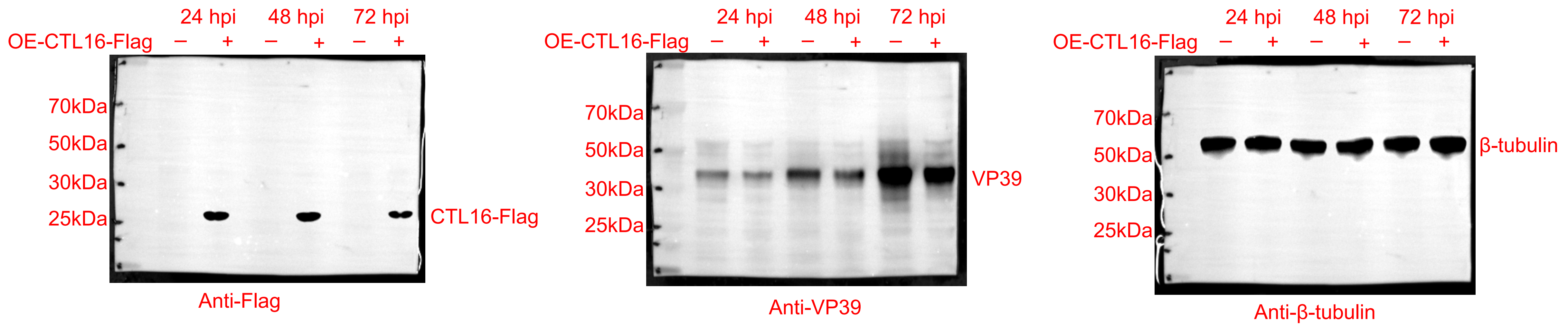

Original Images for Westen Blots Fig 3 D

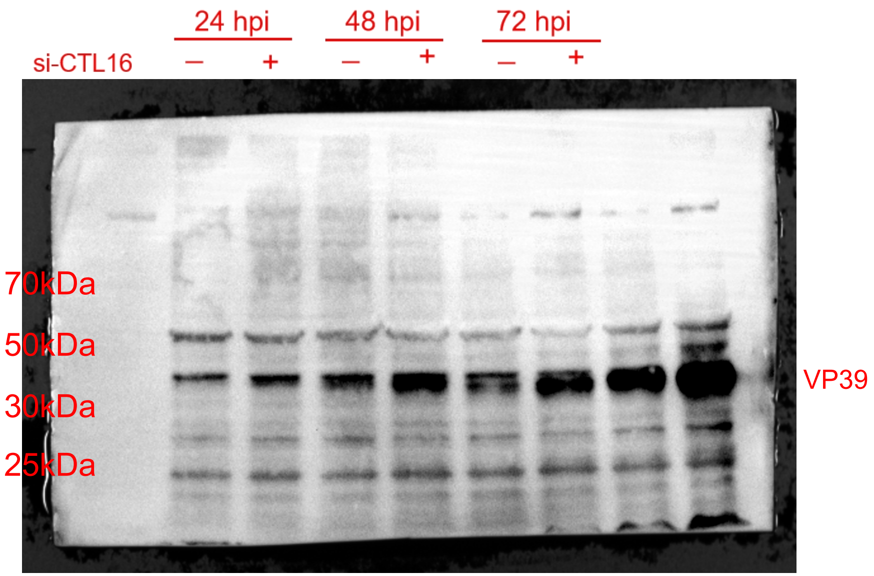

Anti-VP39

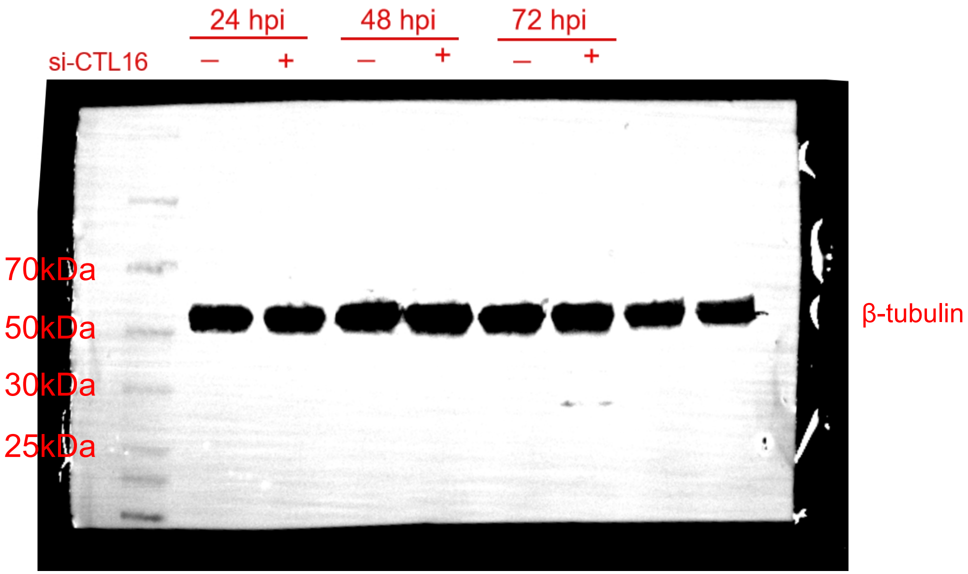

Anti-β-tubulin

Original Images for Westen Blots Fig 4B

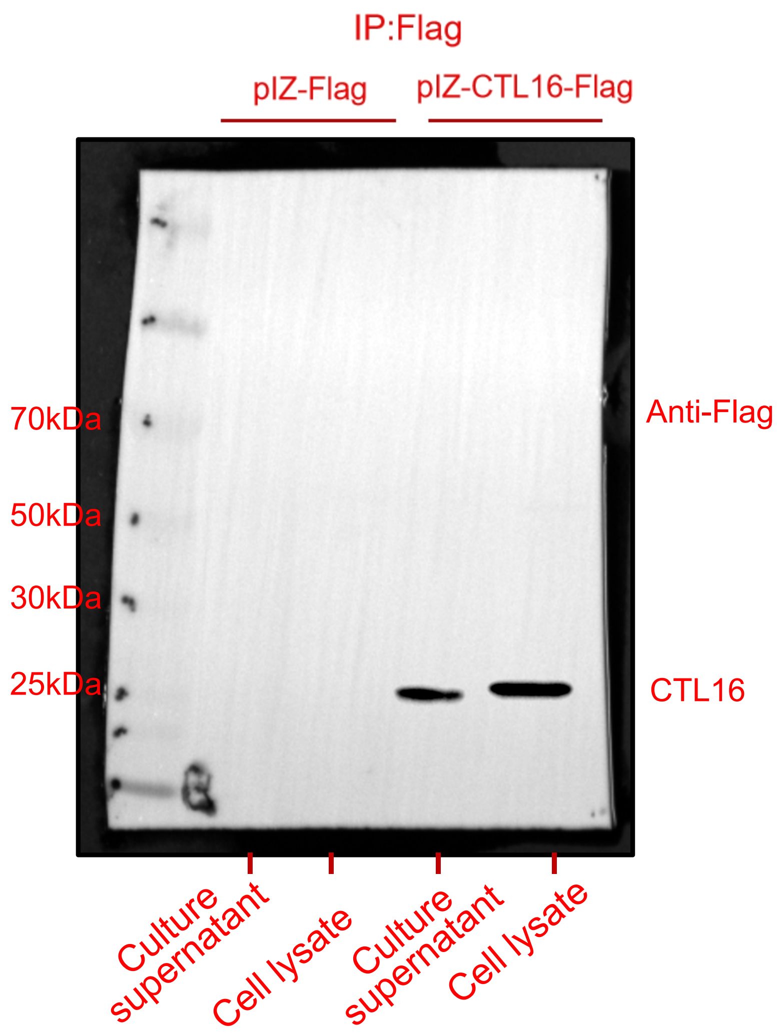

Original Images for Westen Blots Fig 4C

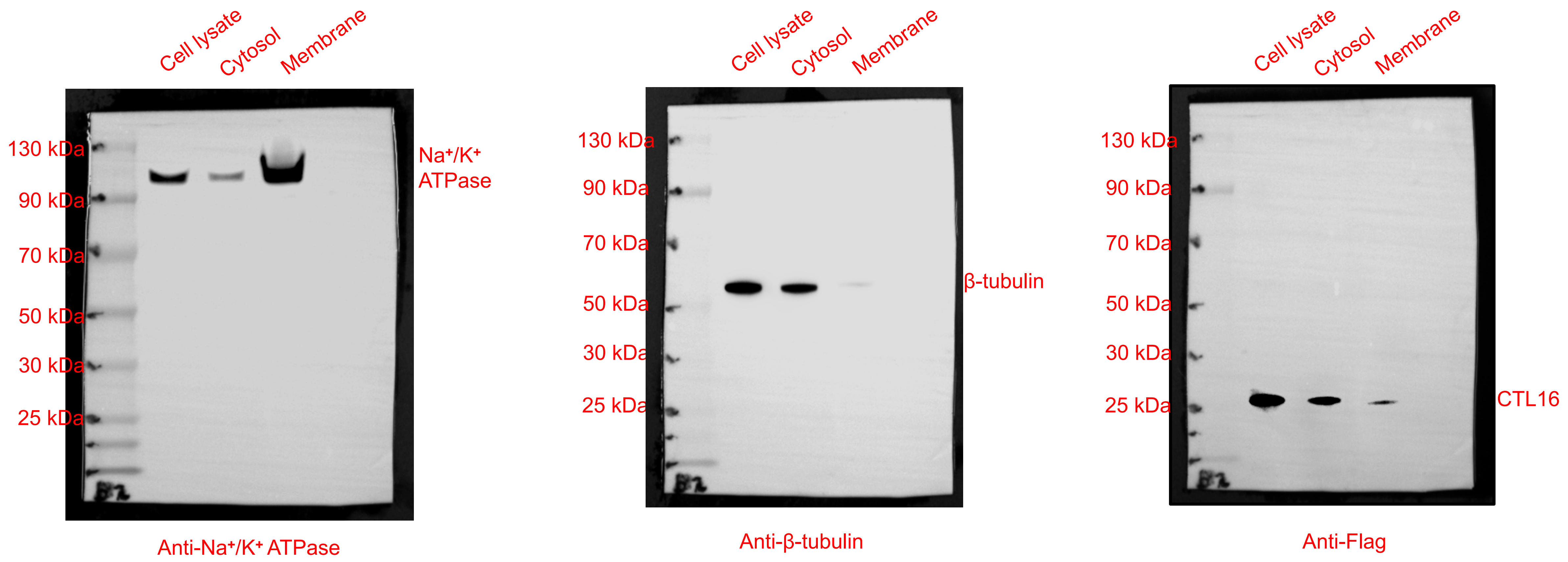

Original Images for Westen Blots Fig 5A

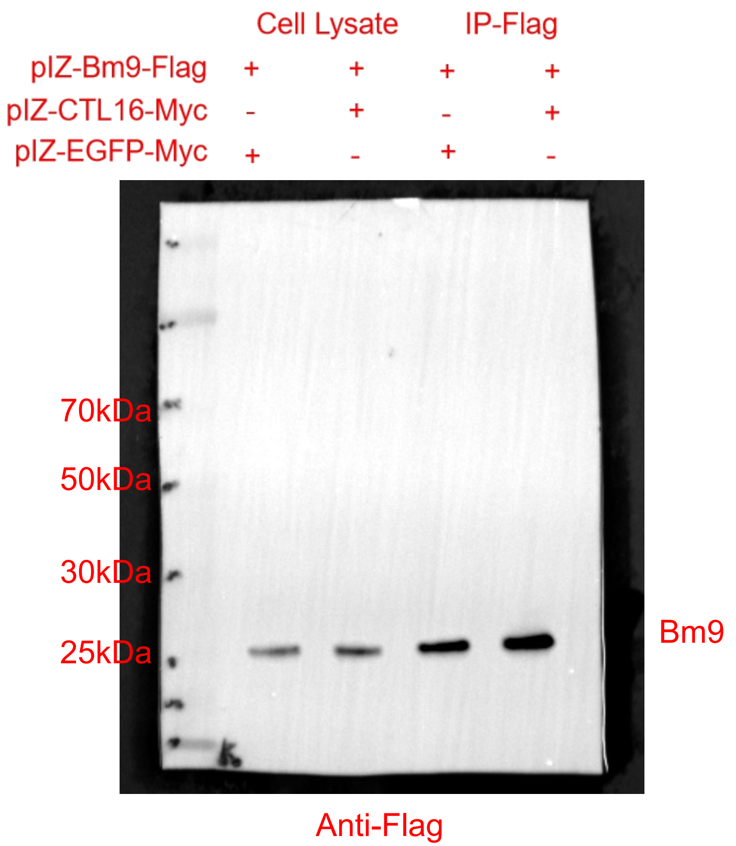

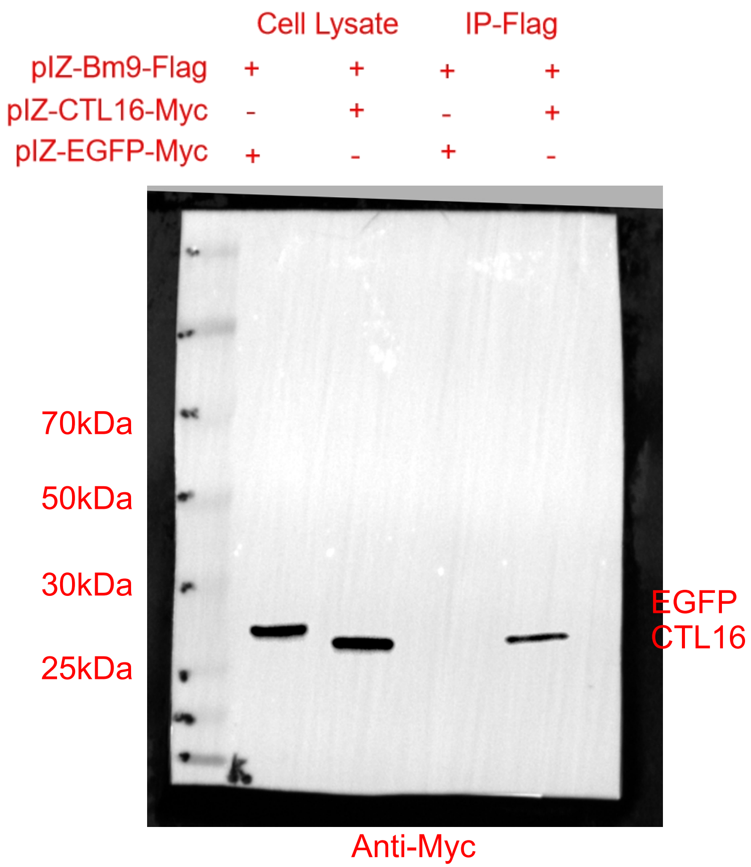

Original Images for Westen Blots Fig 5B

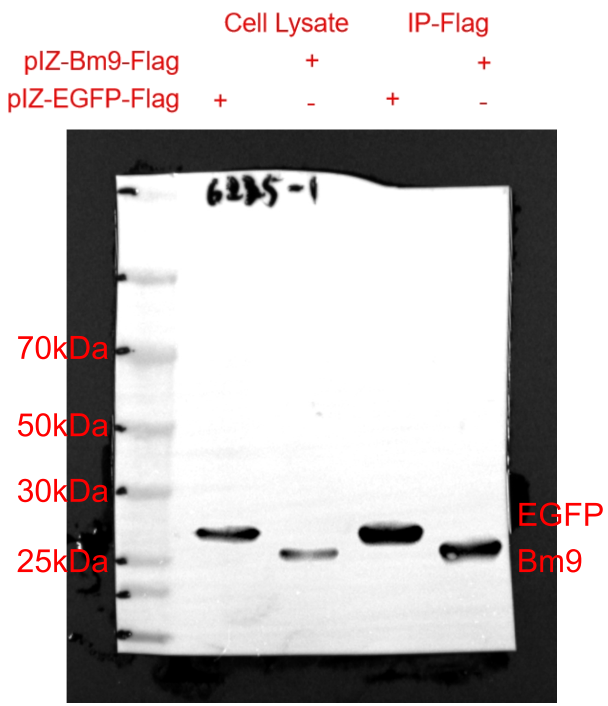

Anti-Flag

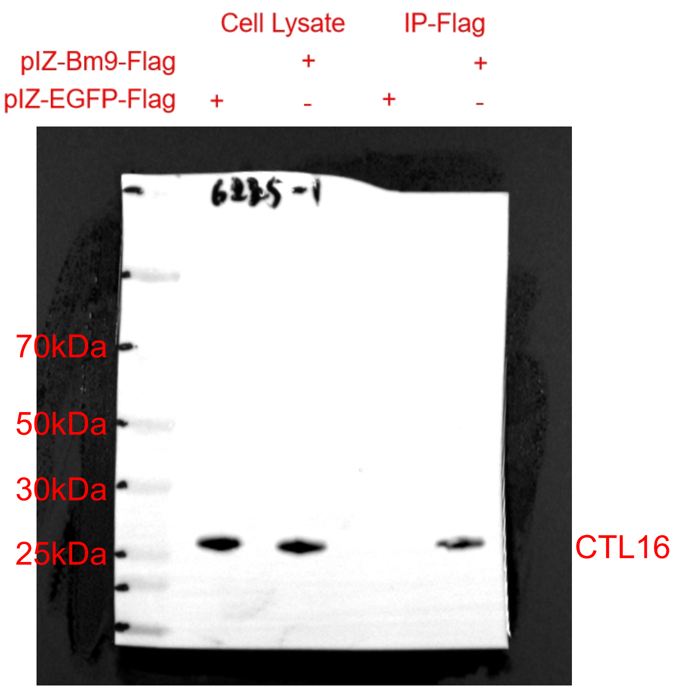

Anti-CTL16

Original Images for Westen Blots Fig 6A

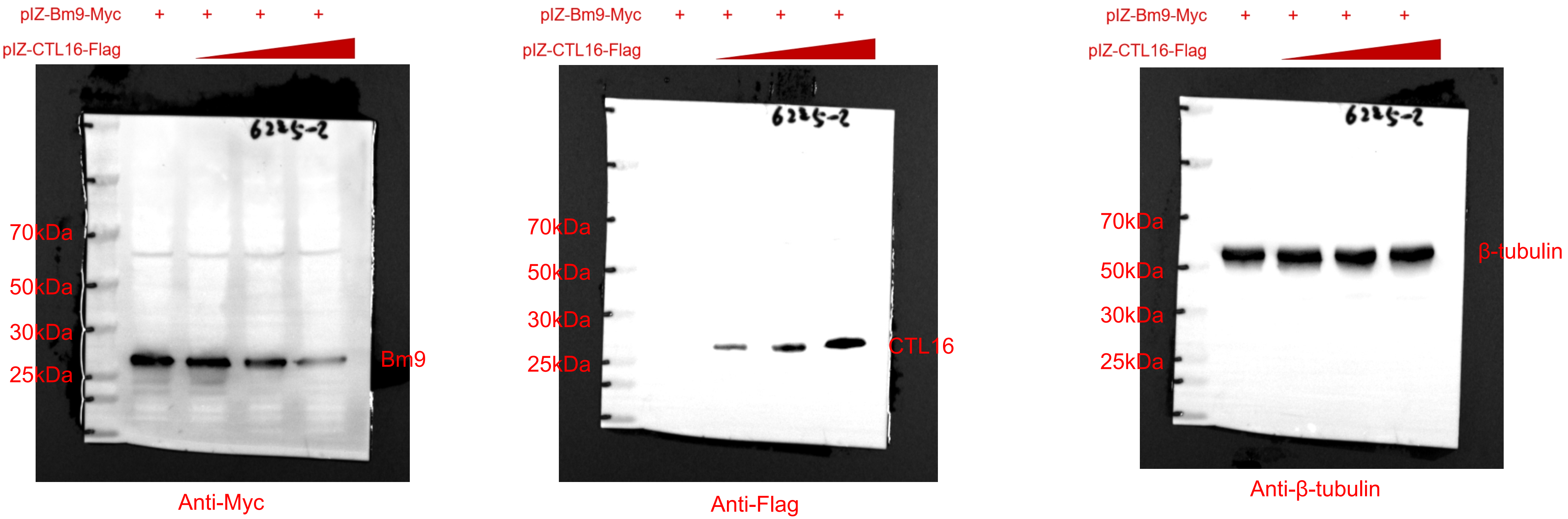

Original Images for Westen Blots Fig 6B

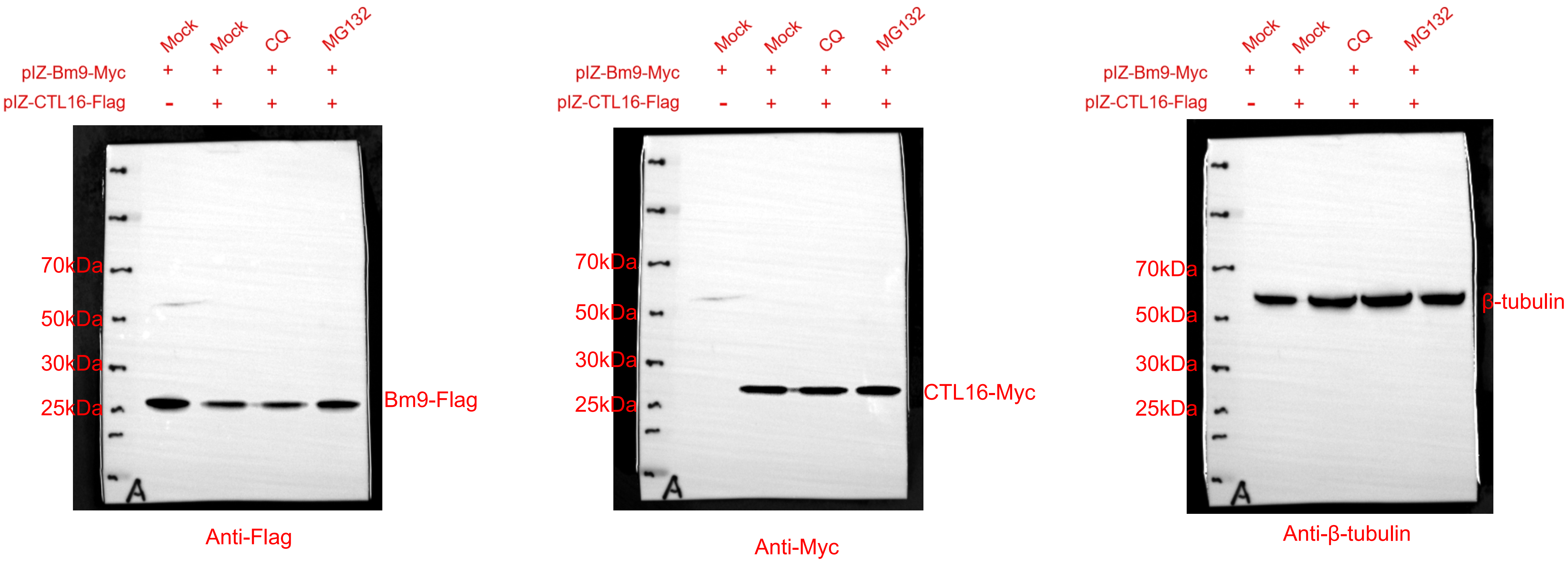

Original Images for Westen Blots Fig 6C

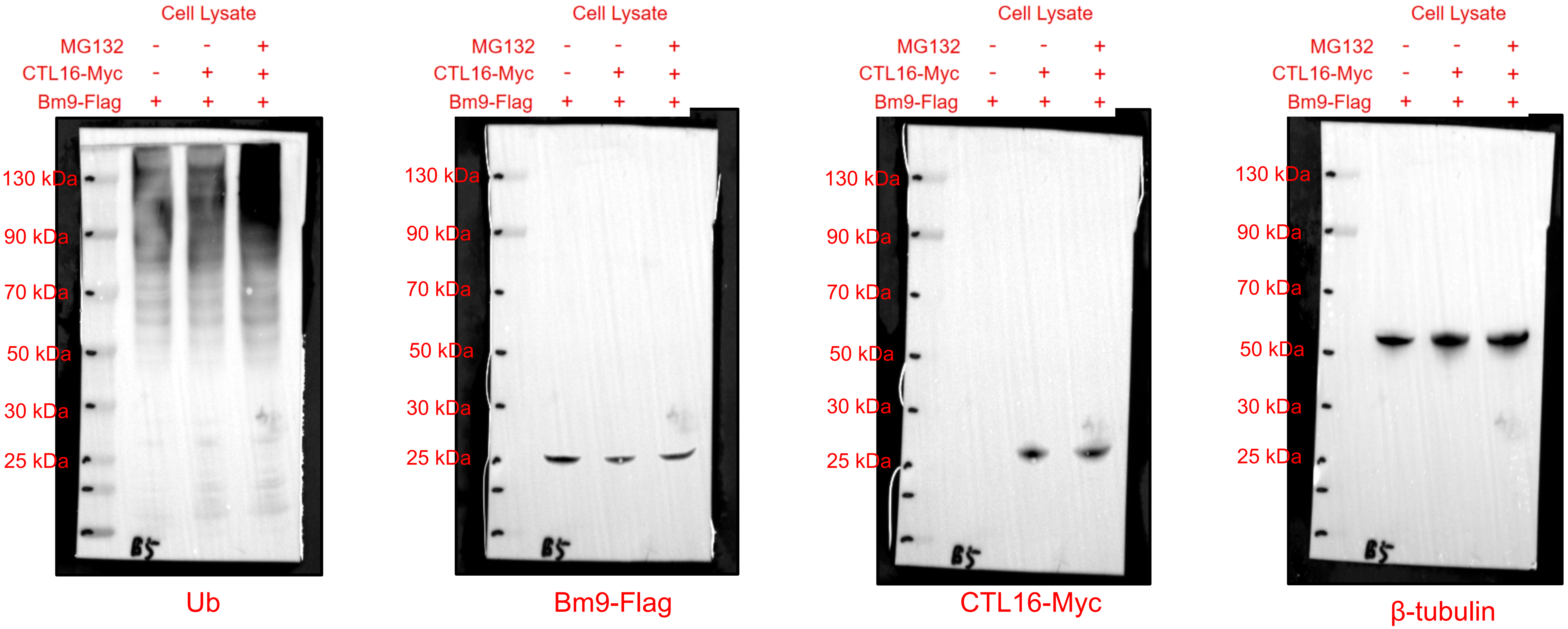

Original Images for Westen Blots Fig 6C

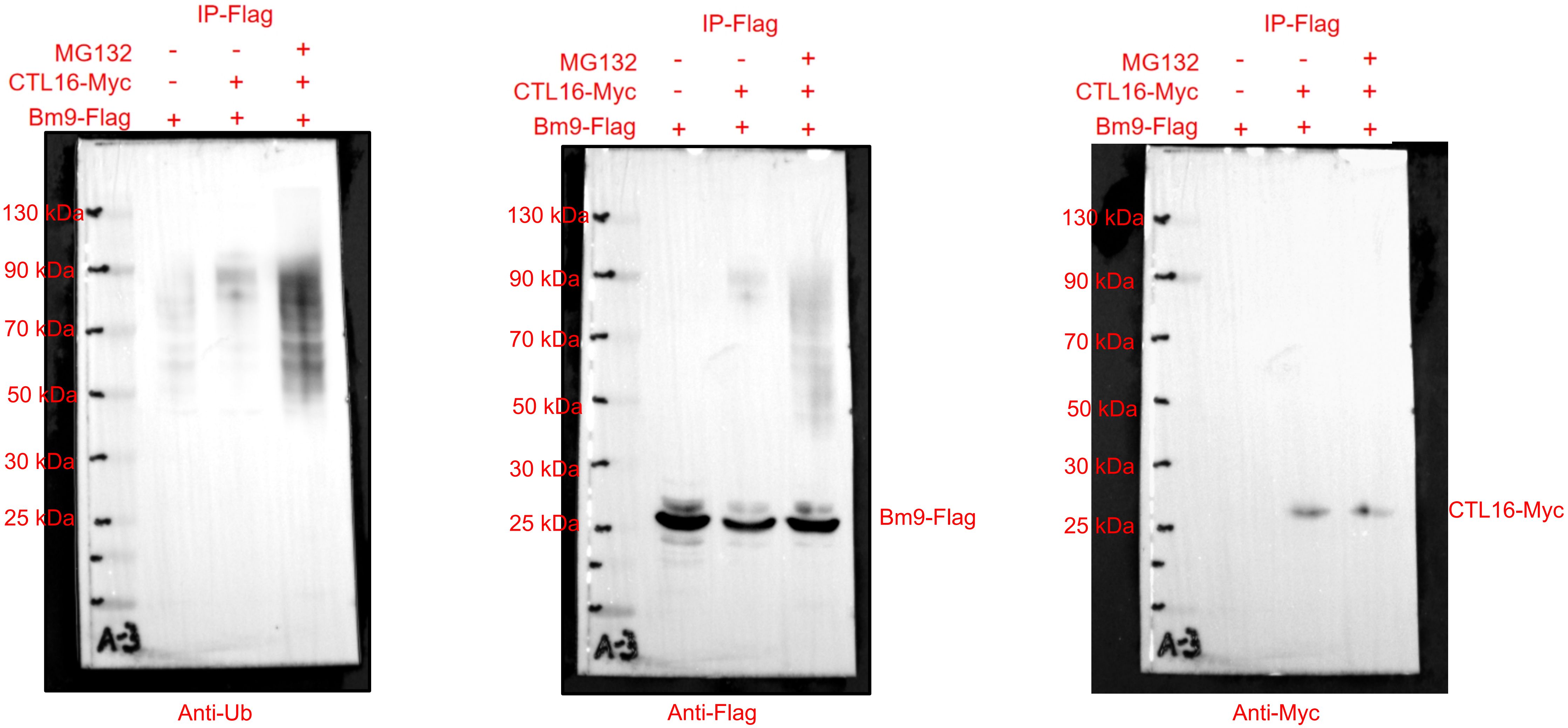

Supplement: Supplementary file 1 [file biomolecules-16-00890-s001.zip › Fig. S1. Original Images for Western Blots.pdf]
