# Supplementary figures and images for: Bombyx mori C-Type Lectin 16 Inhibits BmNPV Proliferation by Degrading Viral Protein Bm9 via Ubiquitin–Proteasome System
Source: Biomolecules. 2026 Jun 17;16(6):890. doi: 10.3390/biom16060890 (PMC13297094; doi:10.3390/biom16060890)

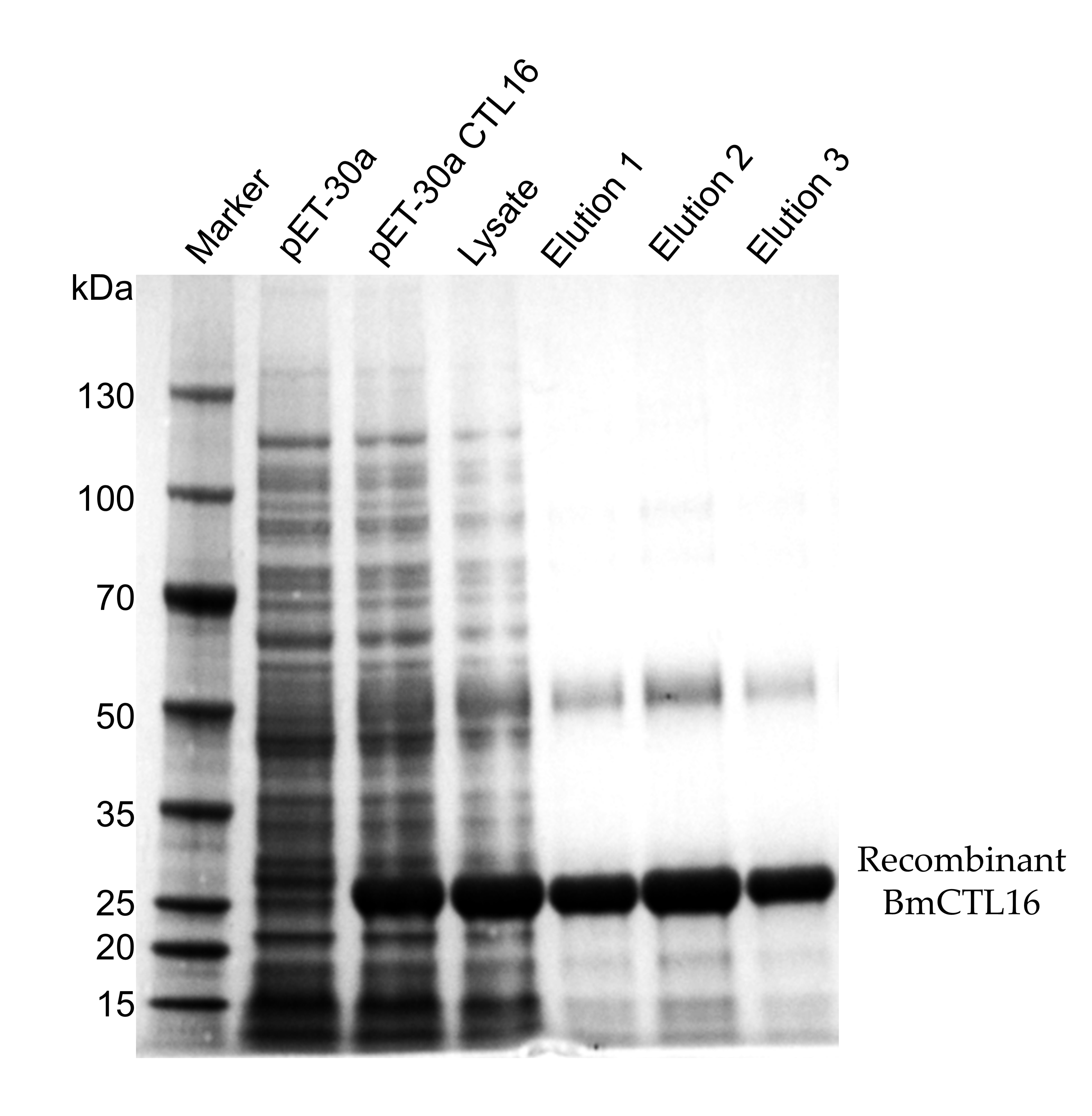

Supplement: Supplementary file 1 [file biomolecules-16-00890-s001.zip › Fig. S2. Expression and purification of recombinant BmCTL16 protein.tif]
